# Impact of Diabetes on Coronary Angiographic Findings in ST-Elevation Myocardial Infarction Patients: A Comparative Study

**DOI:** 10.7759/cureus.91675

**Published:** 2025-09-05

**Authors:** Muzdalfa Parvez, Mudasir Habib, Amjad Ali, Bibi Naila, Noor ul Saba Khattak, Maria Wahab, Kiran Hira, Muhammad Afnan

**Affiliations:** 1 Cardiology, Hayatabad Medical Complex, Peshawar, PAK; 2 Cardiology, Divisional Headquarter Teaching Hospital KDA, Kohat, PAK; 3 Cardiology, Khyber Teaching Hospital, Peshawar, PAK; 4 Internal Medicine, Divisional Headquarter Teaching Hospital KDA, Kohat, PAK; 5 Medicine, Divisional Headquarter Teaching Hospital KDA, Kohat, PAK

**Keywords:** coronary angiography, coronary artery disease, diabetes mellitus, gensini score, multivessel disease, stemi

## Abstract

Introduction: Differences in the angiographic profile of diabetic versus non-diabetic patients with ST-elevation myocardial infarction (STEMI) may influence treatment strategies and outcomes. This study aimed to compare angiographic severity and lesion characteristics between these groups.

Methods: In this prospective comparative cross-sectional study, 150 consecutively enrolled STEMI patients (75 diabetics, 75 non-diabetics) were recruited at Hayatabad Medical Complex (HMC) over 12 months (July 2022-June 2023). Diabetes mellitus (DM) was defined according to American Diabetes Association (ADA) criteria or documented use of glucose-lowering therapy. Inclusion criteria were adults aged 30-80 years presenting with STEMI (per European Society of Cardiology (ESC) guidelines) undergoing primary or early invasive angiography. Exclusion criteria included prior percutaneous coronary intervention (PCI)/coronary artery bypass grafting (CABG), stage IV/V chronic kidney disease, or inadequate angiographic data. Two independent interventional cardiologists, blinded to diabetes status, assessed angiograms for vessel involvement, calcification, diffuse disease, and severity using the Modified Gensini Score. Interobserver agreement was evaluated (κ = 0.87). Statistical analyses included chi-square test and t-test with reporting of 95% confidence intervals (CIs), and logistic regression was used to adjust for confounders (age, hypertension, dyslipidemia, smoking). A priori sample size of 150 was determined to provide 80% power to detect a 15% difference in triple-vessel disease (TVD) prevalence between groups (α = 0.05).

Results: Diabetic patients had a higher prevalence of TVD (30, 40.0% vs. 18, 24.0%; p = 0.031, 95% CI: 1.05-3.81), diffuse disease (41, 54.7% vs. 23, 30.7%; p = 0.003, 95% CI: 1.36-4.87), and significant calcification (29, 38.7% vs. 14, 18.7%; p = 0.007, 95% CI: 1.22-4.56). The mean Gensini score was significantly higher in diabetics (42.6 ± 9.4) than non-diabetics (35.8 ± 8.7; mean difference 6.8, 95% CI: 3.4-10.2, p < 0.001). Adjusted analyses confirmed diabetes as an independent predictor of more severe and complex coronary artery disease (CAD). No significant differences were observed in culprit vessel distribution or thrombolysis in myocardial infarction (TIMI) 3 flow post PCI. At six-month follow-up, diabetics had higher rates of recurrent angina (11, 14.7% vs. 5, 6.7%), though mortality differences were not statistically significant.

Conclusion: STEMI patients with diabetes demonstrate more severe, diffuse, and calcified CAD compared to non-diabetics, even after adjusting for confounders. These findings underscore the need for early, aggressive cardiovascular risk management and tailored interventional strategies in diabetic STEMI populations. Ethical approval was obtained (Ref: 757/CD/HMC/2022), and all patients provided informed consent.

## Introduction

Cardiovascular diseases (CVDs) remain the leading cause of morbidity and mortality worldwide, with acute myocardial infarction (AMI) representing one of the most critical clinical presentations [1]. Among its various forms, ST-elevation myocardial infarction (STEMI) is a life-threatening condition resulting from the complete occlusion of a coronary artery [2]. Prompt recognition and early intervention, particularly via coronary angiography and subsequent revascularization, are essential for improving survival and long-term outcomes [3].

Diabetes mellitus (DM), a metabolic disorder characterized by chronic hyperglycemia, is recognized as a traditional and major risk factor for coronary artery disease (CAD). Evidence from large cohort studies and meta-analyses consistently shows that diabetic patients have a two- to four-fold higher risk of cardiovascular mortality compared to non-diabetics [4]. Beyond atherosclerosis progression, diabetes contributes to endothelial dysfunction, inflammation, and pro-thrombotic states, all of which intensify coronary artery involvement during acute coronary syndromes such as STEMI [5].

Coronary angiography provides critical insights into both the angiographic severity (defined as the extent and burden of coronary stenosis based on validated scoring systems) and the complexity of disease, reflected in features such as diffuse involvement, multi-vessel lesions, and calcification [6]. These angiographic patterns are particularly important in diabetics because they influence procedural difficulty, risk of complications, long-term prognosis, and the selection of revascularization strategies [7]. Thus, investigating how diabetes shapes angiographic findings in STEMI patients is essential to guide tailored interventions and improve outcomes.

Epidemiological data indicate that South Asia, including Pakistan, bears a disproportionately high burden of both diabetes and premature CAD. The prevalence of diabetes in Pakistan is estimated at 16.9%, among the highest globally, while STEMI remains a major contributor to hospital admissions for acute coronary syndromes [8,9]. Despite this, there is a scarcity of regional studies exploring the angiographic patterns of STEMI patients in relation to diabetes, even though such differences may be more pronounced in populations with higher baseline cardiometabolic risk [10].

Although several international studies have demonstrated that diabetic patients often present with more diffuse, calcified, and multi-vessel coronary disease compared to non-diabetics, findings remain inconsistent across different populations and healthcare systems [11,12]. Furthermore, data from low- and middle-income countries are limited, leaving a critical gap in region-specific evidence that could inform therapeutic strategies and resource allocation.

This study was therefore designed to evaluate the angiographic characteristics of diabetic and non-diabetic STEMI patients presenting to a tertiary care hospital in Peshawar, Pakistan. By focusing on a population with a high prevalence of diabetes and cardiovascular risk factors, this work aims to provide locally relevant evidence and address a gap not sufficiently explored in prior international literature.

## Materials and methods

Study design and setting

This comparative cross-sectional study was conducted at the Cardiology Department of Hayatabad Medical Complex (HMC), Peshawar, over a 12-month period, from July 1, 2022, to June 30, 2023. The objective was to compare coronary angiographic findings in STEMI patients with and without DM. Eligible patients were recruited using a non-probability consecutive sampling strategy, ensuring that all patients meeting the criteria during the study period were included. While this approach minimized the risk of selective inclusion, we acknowledge the possibility of sampling bias.

Study population and inclusion criteria

All adult patients aged 30 to 80 years who presented with STEMI and underwent primary or early invasive coronary angiography during the study period were considered eligible. The diagnosis of STEMI was made according to the European Society of Cardiology (ESC) Guidelines for the management of acute coronary syndromes in patients presenting with ST-segment elevation [13]. DM was diagnosed based on American Diabetes Association (ADA) criteria (fasting plasma glucose ≥126 mg/dL, HbA1c ≥6.5%, or random plasma glucose ≥200 mg/dL with symptoms), or documented history of DM with ongoing glucose-lowering therapy. Exclusion criteria included inadequate angiographic information, stage IV or higher chronic kidney disease, and prior coronary interventions (percutaneous coronary intervention (PCI) or coronary artery bypass grafting (CABG)).

Sample-size calculation

Although a similar study with 150 STEMI patients (75 diabetic and 75 non-diabetic) informed the design, a formal power calculation was also performed [14]. This confirmed that a sample size of 150 patients would provide 80% power to detect a 15% difference in the prevalence of triple-vessel disease (TVD) between groups, at a significance level of α = 0.05. This number was therefore adopted to ensure both statistical validity and comparability with prior literature. Eligible patients were adults aged 30-80 years presenting with STEMI and undergoing primary or early invasive angiography, while those with inadequate angiographic data, stage IV or higher chronic kidney disease, or prior PCI/CABG were excluded.

Data-collection procedure

A structured proforma (see Appendix for supplementary data) was used to collect demographic characteristics (age, sex), cardiovascular risk factors (smoking, dyslipidemia, hypertension, family history of CAD), and comorbidities. Coronary angiography was performed via femoral or radial access, following institutional protocols and international guidelines [13,15]. To reduce procedural variability, the angiograms were performed by the same interventional cardiology team. Two experienced cardiologists independently reviewed the films while blinded to diabetes status. Interobserver agreement for angiographic scoring was assessed using kappa statistics (κ = 0.87), confirming strong reliability. Any disagreements were resolved by consensus.

Operational definitions were as follows: diffuse disease was defined as a lesion length >20 mm, calcification as radiopacities visible before contrast injection within the vessel wall, and thrombus burden as angiographic evidence of filling defect, haziness, or embolization. Angiographic severity was quantified using the Modified Gensini Score, which weights the degree and location of stenosis. Parameters assessed included number of diseased vessels (single, double, or triple), culprit vessel (left anterior descending (LAD), right coronary artery (RCA), left circumflex (LCX), or left main), presence of diffuse disease, calcification, thrombus burden, and thrombolysis in myocardial infarction (TIMI) flow grade pre and post PCI.

Data analysis

Data were analyzed using SPSS version 26.0. Continuous variables were expressed as mean ± SD and compared using the Independent Samples t-test. Categorical variables were reported as frequencies and percentages and compared using the chi-square test. Multivariable logistic regression analysis was performed to adjust for potential confounding variables, including age, sex, hypertension, dyslipidemia, and smoking status. Adjusted odds ratios (ORs) with 95% confidence intervals (CIs) were calculated for key angiographic outcomes. P-values less than 0.05 were considered statistically significant. Missing or incomplete records were excluded from the analysis.

Ethical considerations

The study was approved by the institutional review Bboard of HMC (Approval No. 757/CD/HMC/2022, dated June 7, 2022). All participants provided written informed consent. The study complied with the principles of the Declaration of Helsinki, and patient confidentiality was maintained throughout.

## Results

Table 1 shows the baseline clinical and demographic characteristics of STEMI patients with and without diabetes. The group with diabetes had a substantially higher mean age (60.2 ± 9.8 years) than the group without diabetes (56.6 ± 11.1 years, mean difference = 3.6 years, 95% CI: 0.6-6.6, p = 0.020). Hypertension was more common in diabetics (46, 61.3% vs. 31, 41.3%; OR: 2.27, 95% CI: 1.14-4.54, p = 0.017). Smoking was more common in non-diabetics (41, 54.7% vs. 29, 38.7%; OR: 0.52, 95% CI: 0.27-0.99, p = 0.048). There were no significant differences in gender distribution, dyslipidemia, or family history of CAD. These results indicate that diabetic patients with STEMI tend to be older and more hypertensive, while non-diabetics are more likely to be smokers.

**Table 1:** Baseline demographic and clinical characteristics p-value < 0.05 considered statistically significant CAD: Coronary artery disease; CI: Confidence interval; Hx: History

Characteristic	Diabetic (n = 75)	Non-Diabetic (n = 75)	Test Used	Test Value	95% CI	p-value
Age (years, mean ± SD)	60.2 ± 9.8	56.6 ± 11.1	Independent Samples t-test	t = 2.36	0.6–6.6	0.020
Male, n (%)	49 (65.3%)	53 (70.7%)	Chi-square test	χ² = 0.52	—	0.472
Hypertension, n (%)	46 (61.3%)	31 (41.3%)	Chi-square test	χ² = 5.72	1.14–4.54	0.017
Smoking, n (%)	29 (38.7%)	41 (54.7%)	Chi-square test	χ² = 3.93	0.27–0.99	0.048
Dyslipidemia, n (%)	37 (49.3%)	28 (37.3%)	Chi-square test	χ² = 2.22	—	0.136
Family Hx of CAD (%)	18 (24.0%)	20 (26.7%)	Chi-square test	χ² = 0.14	—	0.713

**Figure 1:**
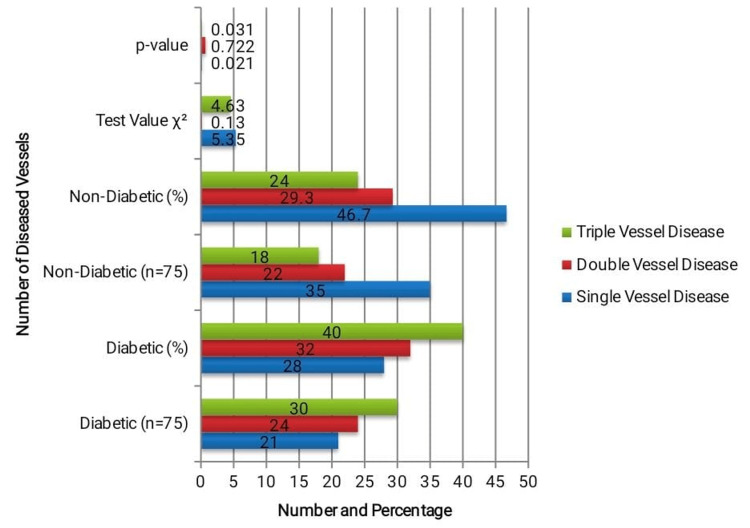
Angiographic severity – number of diseased vessels p < 0.05 statistically significant

The number of significantly diseased coronary vessels was assessed to determine the angiographic severity of CAD, as Illustrated in Figure 1. TVD was significantly more common in diabetic patients (30 (40.0%)), compared to 18 (24.0%) in non-diabetics (χ² = 4.63, p = 0.031). Conversely, single-vessel disease (SVD) was more prevalent in people without diabetes (35 (46.7%)) than diabetics (21 (28.0%)) (χ² = 5.35, p = 0.021). There was no significant difference in the frequency of double-vessel disease (DVD) between the groups. These findings suggest that diabetic STEMI patients present more frequently with multivessel involvement, indicating a more severe disease burden.

**Figure 2:**
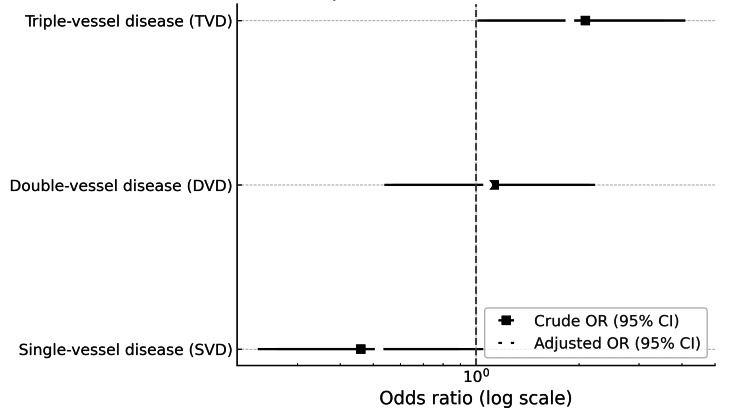
Crude and adjusted ORs with 95% CIs for SVD, DVD, and TVD in diabetic versus non-diabetic STEMI patients The horizontal lines represent CIs, and adjustments were made for age, sex, hypertension, dyslipidemia, and smoking. CI: Confidence interval; DVD: Double-vessel disease; OR: Odds ratio; SVD: Single-vessel disease; STEMI: ST-elevation myocardial infarction; TVD: Triple-vessel disease

Figure 2 illustrates the crude and adjusted ORs with 95% CIs for SVD, DVD, and TVD in diabetic versus non-diabetic STEMI patients, showing that after adjustment for age, sex, hypertension, dyslipidemia, and smoking, diabetes remained independently associated with a higher likelihood of TVD.

**Figure 3:**
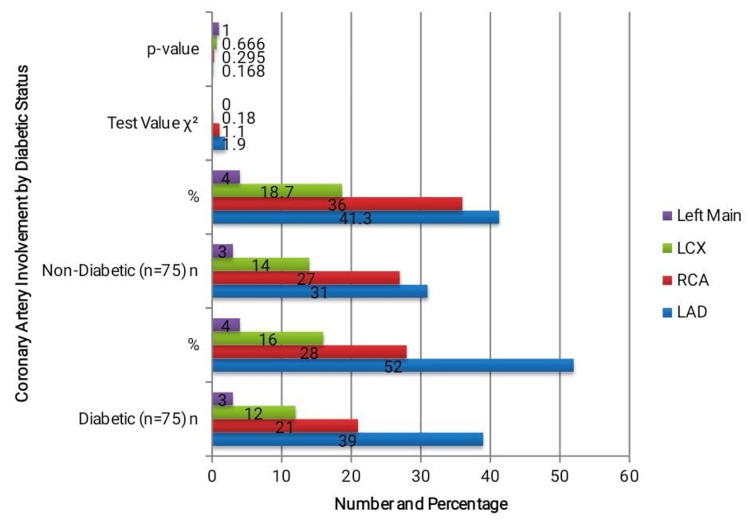
Culprit vessel involvement The table shows culprit vessel distribution in diabetic and non-diabetic STEMI patients, with LAD being most common in both groups. No statistically significant difference was observed (p > 0.05). LAD: Left anterior descending; RCA: Right coronary artery; LCX: Left circumflex

The LAD was the most commonly implicated culprit artery in both groups, as shown in Figure 3 (31 (41.3%) in non-diabetics and 39 (52.0%) in diabetics). Nevertheless, the difference (χ² = 1.90, p = 0.168) was not statistically significant. The second most frequent offender was the RCA, which was detected in 27 (36.0%) non-diabetics and 21 (28.0%) diabetics (p = 0.295). Left main illness and LCX were equally distributed and comparatively uncommon. The total distribution of the guilty vessels did not show any statistically significant differences. These results imply that the distribution of arteries linked to infarcts in STEMI is not substantially changed by diabetes.

As presented in Table 2, diffuse disease was significantly more prevalent among diabetic patients (41 (54.7%)) compared to non-diabetics (23 (30.7%)) (χ² = 8.91, p = 0.003). Similarly, significant coronary calcification was observed in 29 (38.7%) diabetics and 14 (18.7%) non-diabetics (p = 0.007). Non-diabetics had better post-PCI TIMI 3 flow restoration and thrombus burden, but the differences were not statistically significant. These findings demonstrate the more intricate character of CAD, which is marked by widespread involvement and calcification, in diabetic STEMI patients.

**Table 2:** Additional angiographic features CI: Confidence interval; OR: Odds ratio; PCI: Percutaneous coronary intervention; TIMI: Thrombolysis in myocardial infarction

Feature	Diabetic (n = 75)	Non-Diabetic (n = 75)	Test Used	Test Value	95% CI/OR	p-value
Diffuse Disease	41 (54.7%)	23 (30.7%)	Chi-square	χ² = 8.91	OR: 2.72 (1.37–5.40)	0.003
Significant Calcification	29 (38.7%)	14 (18.7%)	Chi-square	χ² = 7.27	OR: 2.74 (1.27–5.91)	0.007
High Thrombus Burden	27 (36.0%)	20 (26.7%)	Chi-square	χ² = 1.62	OR: 1.52 (0.73–3.16)	0.204
TIMI 3 Flow Post-PCI	60 (80.0%)	66 (88.0%)	Chi-square	χ² = 1.73	OR: 0.56 (0.22–1.40)	0.188

The angiographic severity was further quantified using the Modified Gensini Score, as shown in Table 3. Diabetic patients had a significantly higher mean score (42.6 ± 9.4) compared to non-diabetics (35.8 ± 8.7) (t = 4.64, p < 0.001). Additionally, 49 (65.3%) diabetics had a score ≥ 40 versus only 28 (37.3%) non-diabetics (χ² = 11.15, p = 0.001). These results clearly imply that in STEMI patients, diabetes is linked to increased angiographic burden and CAD severity.

**Table 3:** Angiographic severity score (Modified Gensini Score) Modified Gensini Score estimates angiographic disease burden. The results were statistically significant (p < 0.05). CI: Confidence interval; OR: Odds ratio

Variable	Diabetic (n = 75)	Non-Diabetic (n = 75)	Test Used	Test Value	95% CI/Effect Size	p-value
Mean Gensini Score ± SD	42.6 ± 9.4	35.8 ± 8.7	Independent Samples t-test	t = 4.64	6.8 (3.4–10.2)	<0.001
Score ≥ 40, n (%)	49 (65.3%)	28 (37.3%)	Chi-square test	χ² = 11.15	OR: 3.19 (1.57–6.50)	0.001

## Discussion

The effect of DM on coronary angiographic results in individuals who present with STEMI was investigated in this study. The results showed that diabetic patients had significantly more extensive and severe CAD than non-diabetics. Notably, diabetic patients were more likely to have TVD, higher Modified Gensini scores, diffuse atherosclerosis, and coronary calcification. Although the distribution of culprit vessels and post-procedural TIMI flow showed no significant differences, diabetics exhibited a more complex angiographic profile overall. These findings carry important clinical implications, as higher Gensini scores and multivessel disease are associated with more complex revascularization procedures and potentially poorer prognoses.

When compared with existing studies, the present findings align closely with previously reported trends [16]. Diabetic patients consistently exhibit multivessel disease more frequently than non-diabetics, reflecting the systemic and progressive nature of atherosclerosis in diabetes [17]. Higher angiographic severity scores and the prevalence of diffuse and calcified lesions among diabetics are also commonly reported [18]. At the same time, some contrasting studies have shown less pronounced differences, highlighting variability across geographic and socioeconomic contexts. By situating our findings alongside both consistent and divergent reports, we avoid selective reporting and emphasize the importance of population-specific research. These findings reinforce the notion that hyperglycemia, insulin resistance, and associated metabolic disturbances accelerate endothelial dysfunction and plaque formation, leading to more complex coronary lesions [19].

The increased incidence of hypertension and older age among diabetics in the current cohort matches demographic patterns observed in prior research [20]. While these baseline imbalances may partly explain the differences in angiographic severity, our adjusted analyses confirmed that diabetes remained an independent predictor. The lack of significant difference in culprit vessel distribution also aligns with most previous angiographic studies, suggesting that while lesion complexity is greater in diabetics, the pattern of acute infarction does not differ significantly [21]. Furthermore, the relatively lower TIMI 3 flow post PCI in diabetics, although not statistically significant, is consistent with known microvascular dysfunction, chronic inflammation, and advanced glycation end-products that impair myocardial perfusion in this group [22].

Limitations must be acknowledged. This study was based on a non-probability consecutive sample at a single tertiary care center, which may limit generalizability and introduce selection bias. Although interobserver variability was minimized by blinded dual review with high kappa agreement, some degree of interpretation bias cannot be excluded. While we adjusted for major confounders (age, hypertension, dyslipidemia, smoking), other variables such as medication history (e.g., statins, antiplatelets, insulin therapy) and duration or control of diabetes (HbA1c levels) were not collected and may have influenced angiographic severity. The absence of long-term outcome data also precludes definitive prognostic conclusions.

Future studies should adopt prospective multicenter cohort designs to evaluate temporal relationships and outcomes in greater detail. Subgroup analyses based on sex, age, duration of diabetes, and glycemic control would help refine risk stratification. Incorporating intravascular imaging modalities, such as intravenous urography (IVU) and optical coherence tomography (OCT), could provide more robust plaque characterization, while linking angiographic patterns to clinical outcomes (e.g., repeat revascularization, mortality) would enhance the translational value of such research.

## Conclusions

This study demonstrated that STEMI patients with diabetes present with more severe and complex CAD compared to non-diabetic patients, characterized by higher angiographic severity scores, greater calcification, diffuse disease, and multivessel involvement. These findings should be interpreted with caution given the single-center design, moderate sample size, and lack of long-term follow-up, but they provide important insights into the angiographic burden of diabetic STEMI patients.

From a clinical perspective, these results highlight the need for early risk identification, aggressive risk factor management, and tailored interventional strategies. In practical terms, personalized interventional methods may include earlier referral for CABG in cases of extensive multivessel disease, greater use of intravascular imaging to guide percutaneous interventions, and integration of angiographic predictors with established clinical risk scores to better inform treatment planning. By recognizing these angiographic patterns, clinicians can optimize revascularization decisions and strengthen secondary prevention strategies for this high-risk population.

## Appendices

**Table 4:** Data collection proforma CAD: Coronary artery disease; DVD: Double-vessel disease; HDL: High-density lipoprotein; LAD: Left anterior descending; LCX: Left circumflex; LDL: Low-density lipoprotein; RCA: Right coronary artery; SVD: Single-vessel disease; TIMI: Thrombolysis in myocardial infarction; TVD: Triple-vessel disease

Section	Variables Collected
Demographics	Age, Sex
Medical History	Diabetes Mellitus (Yes/No), Hypertension (Yes/No), Family History of CAD (Yes/No)
Lifestyle Factors	Smoking Status (Current/Former/Never)
Laboratory Parameters	Lipid Profile (Total Cholesterol, LDL, HDL, Triglycerides)
Angiographic Findings	Access Site (Femoral/Radial), Number of Diseased Vessels (SVD/DVD/TVD)
	Culprit Vessel (LAD, RCA, LCX, Left Main)
	Presence of Diffuse Disease (Yes/No)
	Presence of Calcification (Yes/No)
	Thrombus Burden (Mild/Moderate/Severe)
	TIMI Flow Grade Pre and Post Intervention (Grade 0–3)
Assessment Details	Interpreted by Two Independent Interventional Cardiologists (Blinded to Diabetic Status)

## References

[1] Młynarska E, Czarnik W, Fularski P (2024). From atherosclerotic plaque to myocardial infarction - the leading cause of coronary artery occlusion. Int J Mol Sci.

[2] Elendu C, Amaechi DC, Elendu TC (2023). Comprehensive review of ST-segment elevation myocardial infarction: Understanding pathophysiology, diagnostic strategies, and current treatment approaches. Medicine (Baltimore).

[3] Akbar H, Mountfort S (2024). Acute ST-Segment Elevation Myocardial Infarction (STEMI). StatPearls.

[4] Aronson D, Edelman ER (2014). Coronary artery disease and diabetes mellitus. Cardiol Clin.

[5] Ye J, Li L, Wang M (2022). Diabetes mellitus promotes the development of atherosclerosis: the role of NLRP3. Front Immunol.

[6] Zhang HW, Jin JL, Cao YX (2021). Association of diabetes mellitus with clinical outcomes in patients with different coronary artery stenosis. Cardiovasc Diabetol.

[7] Hussain S, Zaman S, Khan MA, Khan I, Iftekhar MF (2024). Comparison of angiographic success and clinical outcomes based on different percutaneous coronary intervention techniques. Cureus.

[8] Sharma V, Sharma K, Mansuri Z, Jain S, Bhatia S, Patel K (2020). Does angiographic profile and outcome of diabetic patients among Asian Indians correlate with presenting glycated hemoglobin during acute ST-elevation myocardial infarction? DECIPHER study. J Pract Cardiovasc Sci.

[9] Wu TG, Wang L (2002). Angiographic characteristics of the coronary artery in patients with type 2 diabetes. Exp Clin Cardiol.

[10] Bettamer Z, Elkadiki AH, Alsaeiti KD (2021). Coronary angiographic characteristics of type 2 DM compared with nondiabetic patients in Benghazi-Libya. A cross-sectional study. Libyan J Med Sci.

[11] Flood D, Seiglie JA, Dunn M (2021). The state of diabetes treatment coverage in 55 low-income and middle-income countries: a cross-sectional study of nationally representative, individual-level data in 680 102 adults. Lancet.

[12] Agrawal A, Lamichhane P, Eghbali M (2023). Risk factors, lab parameters, angiographic characteristics and outcomes of coronary artery disease in young South Asian patients: a systematic review. J Int Med Res.

[13] Ibanez B, James S, Agewall S (2018). 2017 ESC Guidelines for the management of acute myocardial infarction in patients presenting with ST-segment elevation: The Task Force for the management of acute myocardial infarction in patients presenting with ST-segment elevation of the European Society of Cardiology (ESC). Eur Heart J.

[14] Pathak SR, Gajurel RM, Poudel CM, Shrestha H, Thapa S, Thapa S, Koirala P (2021). Angiographic severity of coronary artery disease in diabetic and non-diabetic acute STEMI patients in a tertiary care centre of Nepal. Kathmandu Univ Med J.

[15] O'Gara PT, Kushner FG, Ascheim DD (2013). 2013 ACCF/AHA guideline for the management of ST-elevation myocardial infarction: a report of the American College of Cardiology Foundation/American Heart Association Task Force on Practice Guidelines. J Am Coll Cardiol.

[16] Elbendary MA, Saleh MA, Sabet SS, Bastawy I (2022). Correlation between endothelial dysfunction and occurrence of no-reflow in patients undergoing post-thrombolysis early invasive percutaneous intervention for ST-elevation myocardial infarction. Egypt Heart J.

[17] Qamar A, Bhatia K, Arora S (2023). Clinical profiles, outcomes, and sex differences of patients with STEMI: findings from the NORIN-STEMI Registry. JACC Asia.

[18] Babes EE, Bustea C, Behl T (2022). Acute coronary syndromes in diabetic patients, outcome, revascularization, and antithrombotic therapy. Biomed Pharmacother.

[19] Elakabawi K, Huang X, Shah SA, Ullah H, Mintz GS, Yuan Z, Guo N (2020). Predictors of suboptimal coronary blood flow after primary angioplasty and its implications on short-term outcomes in patients with acute anterior STEMI. BMC Cardiovasc Disord.

[20] Shemesh E, Zafrir B (2019). Coronary angiography in the very old: impact of diabetes on long-term revascularization and mortality. J Geriatr Cardiol.

[21] Hasdai D, Granger CB, Srivatsa SS (2000). Diabetes mellitus and outcome after primary coronary angioplasty for acute myocardial infarction: lessons from the GUSTO-IIb angioplasty substudy. J Am Coll Cardiol.

[22] Dziewierz A, Zdzierak B, Malinowski KP (2022). Diabetes mellitus is still a strong predictor of periprocedural outcomes of primary percutaneous coronary interventions in patients presenting with ST-segment elevation myocardial infarction (from the ORPKI Polish National Registry). J Clin Med.

